# Engaging with faith groups to prevent VAWG in conflict-affected communities: results from two community surveys in the DRC

**DOI:** 10.1186/s12914-020-00246-8

**Published:** 2020-10-07

**Authors:** Elisabet Le Roux, Julienne Corboz, Nigel Scott, Maggie Sandilands, Uwezo Baghuma Lele, Elena Bezzolato, Rachel Jewkes

**Affiliations:** 1 Stellenbosch University, 171 Dorp Street, Stellenbosch, Western Cape 7600 South Africa; 2 Independent Research Consultant, Barcelona, Spain; 3 Gamos Ltd., Crown House, 231 Kings Road, Reading, RG1 4LS UK; 4 Independent Research Consultant, London, UK; 5 Tearfund, 100 Church Road, Teddington, TW11 8QE UK; 6 South African Medical Research Council, 1 Soutpansberg Road, Pretoria, South Africa

**Keywords:** Violence against women and girls, Faith, Religion, Democratic Republic of Congo, Sexual violence, Intimate partner violence, Conflict-affected communities

## Abstract

**Background:**

An evaluation was conducted of a three-year intervention focused on violence against women and girls (VAWG) and implemented in the conflict-affected north-east of the Democratic Republic of Congo (DRC), a country with high rates of VAWG. The intervention addressed VAWG, and especially sexual violence, by specifically engaging with communities of faith and their leaders.

**Methods:**

Two community surveys were conducted, one before and one after the intervention, in three health areas in Ituri Province in the DRC. At both baseline and endline, data was collected from male and female members of randomly selected households in 15 villages (five per health area) in which the intervention was being implemented. At baseline the sample comprised 751 respondents (387 women, 364 men) and at endline 1198 respondents (601 women, 597 men). Questionnaires were interviewer-administered, with sensitive questions related to experience or perpetration of violence self-completed by participants.

**Results:**

The study showed significantly more equitable gender attitudes and less tolerance for IPV at endline. Positive attitude change was not limited to those actively engaged within faith communities, with a positive shift across the entire community in terms of gender attitudes, rape myths and rape stigma scores, regardless of level of faith engagement. There was a significant decline in all aspects of IPV in the communities who experienced the intervention. While the experience and perpetration of IPV reported at endline did not track with exposure to the intervention, it is plausible that in a context where social norm change was sought, the impact of the intervention on those exposed could have had an impact on the behaviour of the unexposed.

**Conclusion:**

This intervention was premised on the assumption that faith leaders and faith communities are a key entry point into an entire community, able to influence an entire community. Research has affirmed this assumption and engaging with faith leaders and faith communities can thus be a strategic intervention strategy. While we are confident of the link between the social norms change and faith engagement and project exposure, the link between IPV reduction and faith engagement and project exposure needs more research.

## Background

Though the First Congo War (1996–1997) and the Second Congo War (1998–2003) have long come to an end, violence continues in the Democratic Republic of Congo (DRC). Characterised by the involvement of various rebel groups and militias, estimates vary, but at least 5 million people have died in this protracted conflict and millions have been displaced [1, 2].

Internationally, the DRC has become synonymous with high rates of sexual violence [3]. Some argue that violence against women and girls (VAWG), including sexual violence, has always been part of Congolese culture, in a society characterised by gender inequality and with customary law dictating that perpetrators need only pay compensation [4, 5]. However, especially sexual violence has increased since the start of the armed conflicts, perpetrated by rebels, militia, soldiers, peacekeepers, and civilians alike [4–7]. Sexual violence appears to have a particularly violent and torturous dimension in this conflict context, as evidenced by acts such as gang rape, forced incest, mutilation of genitals, and abduction as sex slaves [4, 8, 9].

The prevalence of VAWG in the DRC remains unclear, not least of all as conflict is ongoing and hampers reporting and the implementation of studies that attempt to assess the prevalence of VAWG. The 2014 Demographic and Health Survey found that 57% of ever married women aged 15–49 years had ever experienced intimate partner violence (IPV) and 16% had experienced sexual violence in the 12 months prior to the survey [10]. Promundo’s IMAGE study found that 45% of women in the Eastern DRC reported having ever experienced physical IPV and 49% having ever experienced sexual IPV. Rape as part of conflict was reported by 22% of women [11].

The role of faith, faith leaders and faith communities in VAWG is contentious. Some religious beliefs and practices make it harder to cope and integrate traumatic experiences [12, 13]. Furthermore, faith and faith institutions have been blamed for often perpetuating the unequal gender constructs, stigma and discrimination that contributes to the perpetration and normalisation of VAWG, as well as for ostracizing VAWG survivors, especially survivors of sexual violence [12, 14–16]. With “patriarchy ha(ving) God on its side” [17], religion is recognised by some as being a key patriarchal structure within society. The vast majority of African religions and religious institutions, influencing culture but also products of culture, do not accept women’s autonomy, and enforce beliefs, practices and traditions that empower men to the detriment of women [18]. In a study of African church responses to sexual violence against women (SVAW) in the DRC, Rwanda and Liberia, Le Roux [19] found that churches are key patriarchal institutions and that this limits their ability to respond to SVAW, for:

*the ability of churches to address issues that cause instability is limited when the causes are practices and beliefs that lie at the heart of the religion and the institution, especially if these practices and beliefs are upholding the power of those currently in power* [19]*.*

On the other hand, faith, faith leaders and faith communities have tremendous potential to be influential in addressing VAWG. Faith communities have been critical to service delivery in conflict-affected settings, particularly in the areas of health and education [20]. Especially for poor and marginalised people, the social capital produced by faith communities become a key institution providing the emotional, spiritual and physical resources that enable their survival and welfare [21]. Furthermore, religion has the ability to socialise the individual and create group cohesion through common beliefs and value systems [22]. Faith leaders are influential gatekeepers in their communities, with the ability to influence the beliefs and behaviours of their followers [14, 23]. Believers turn to their faith leaders and faith communities in challenging times. Various studies have shown how sexual violence survivors see their faith, faith leader and faith communities as having the ability to provide the support and solace that they need in order to deal with what happened to them [12, 14, 16, 19, 24]. Even if survivors are not receiving it, they believe that it can and should be provided.

The Congolese are a religious people, with atheism and agnosticism extremely rare. The majority of the population is Christian (79% of the population, or even 90% according to some sources) or Muslim (9%) [25]. Religious institutions are some of the few remaining functioning institutions in the DRC – especially in the eastern DRC. Religious networks remain influential in the public sphere and are the biggest sector of civil society [26]. With the majority of Congolese being Christian, churches “wield enormous influence in the public space as providers of social services in a polity that has been characterised by years of misrule, declining state capacity and protracted conflict” [26]. This influence has been used in relation to VAWG. For example, the Anglican Church has implemented programming on the human immune--deficiency virus and sexual and gender-based violence (SGBV) [27], while the World Council of Churches, an international ecumenical fellowship to whom many Congolese churches belong, formally acknowledged and condemned churches’ complicit role in sexual violence and their refusal to address the issue [28].

Unfortunately, it remains the norm for religious institutions to *not* get involved in VAWG. They mostly remain silent and unengaged on the issue. Many continue to promote and condone patriarchal beliefs and practices that lead to the perpetration of VAWG and the stigmatisation of VAWG survivors. This is especially the case in relation to sexual violence, where the cultural taboo on talking about sex and sex-related matters is also present within religious institutions [19].

It is within this context that Tearfund, a Christian relief and development charity, with partner HEAL Africa, a Christian Congolese development organisation, launched a three-year intervention focused on addressing VAWG, and especially sexual violence, by specifically engaging with communities of faith and their leaders. This paper discusses results from the evaluation of this intervention, based on community survey data collected at two evaluation points, baseline and endline. The analysis presented in the paper focuses on a number of key outcomes, including social norms, attitudes associated with violence against women, and experience or perpetration of IPV, especially in relation to faith engagement and exposure to intervention activities.

## Overview of the intervention

Tearfund’s project, ‘Engaging with Faith Groups to Prevent Violence Against Women and Girls in Conflict-affected Communities’ was funded by UK Aid from the UK government, under the *What Works to Prevent Violence Against Women and Girls?* Global Programme. In this project, Tearfund worked with local partner HEAL Africa in remote and conflict-affected communities. The aim of the intervention was to mobilise, train and equip faith leaders to become catalysts within their own communities, in order to address the underlying root causes of VAWG from a faith perspective.

The intervention was implemented across 15 villages with a total population (at baseline) of approximately 13,000 people in 2600 households. Seventy-five faith leaders (five per community) formed part of the intervention, and 30 gender champions (two per community). Faith leaders were from any faith or denomination (Christian or Muslim); gender champions were community leaders, such as midwives or teachers, who showed a willingness to address gender-related matters. Both faith leaders and gender champions were community members (male and female), selected by the Project Manager and Project Officer in consultation with community gatekeepers in each community (e.g. chiefs, clinic staff, government employees, church and mosque leaders, etc.). Faith leaders and gender champions were selected based on actions or attitudes that showed that they were (at least to some extent) opposed to VAWG and/or in favour of gender equality.

Faith leaders and gender champions were trained to address harmful attitudes, behaviours and social norms which support gender inequality and enable VAWG, and received ongoing mentoring from project staff. They were encouraged to act as role models and to incorporate what they have learned into their existing activities such as sermons, prayer groups, youth groups, and counselling, and thus promote gender equality and non-violence throughout the rest of the community. The project used Tearfund’s ‘Transforming Masculinities’ approach, a process for supporting individual and community-wide change on gender violence. Through workshops or structured small group discussions, the process draws on sacred texts to guide reflection on gender equality and positive masculinities. Themes include understanding GBV and how it affects everyone, addressing unequal power and privilege, and discussing positive masculinities. The training materials are available in the public domain.^1^ The selected faith leaders and gender champions received a three-day training in August 2015 entitled “Transforming Masculinities”. Faith leaders and gender champions received the same training, with an additional module on facilitation for the gender champions. Refresher trainings took place in March 2016 (1 day) and September 2016 (1 day). In March 2017 a training was done on psychosocial support (3 days), and in April 2017 a training on counselling and mediation (3 days). However, due to funding challenges the 2017 trainings were only done with selected faith leaders (and no gender champions). This is as faith leaders in this setting are often called upon to provide family mediation and lay counselling for couples or individuals. The training thus aimed to strengthen their lay counselling skills, especially to enhance their ability to support survivors. The training included skills development, such as active listening. All faith leaders and gender champions receive monthly mentoring and monitoring visits from the Project Officer. Furthermore, the Healing of Memories process was conducted with 24 survivors and 9 members from the communities, in July 2015. This was an activity focused specifically on survivors of VAWG (mostly IPV, including sexual violence). The community members included in this workshop were those already involved in supporting survivors, such as family members and faith leaders. The Healing of Memories workshop is experiential, focusing on participants’ emotions and encouraging self-awareness. It facilitates the sharing of life stories, to enable participants to move towards individual emotional healing and mutual understanding. While originally developed in South Africa to address national reconciliation, the workshops are now used in varying contexts, including with sexual violence survivors [29]. The workshop was presented by a consultant, with part of the process focussed on establishing peer support groups. The two approaches – Transforming Masculinities and Healing of Memories – are complementary. As part of the project’s focus on survivors, the Healing of Memories process was meant to happen again at endline, but had to be cancelled due to renewed outbreak of conflict.

Both kinds of trained actors (faith leaders and gender champions) committed to and were expected to use and disseminate what they had learnt during the training. The faith leaders were expected to integrate the learning in their ongoing activities, particularly in public speaking/preaching and couple counselling. Their integration and implementation was monitored (through self-reporting and external monitoring by project staff). The gender champions were entrusted with the delivery of community dialogues, a process that was monitored and supervised by project staff, and also encouraged to integrate the learning wherever possible in their daily activities (e.g. family life, work space, etc).

Through the group discussions facilitated by the gender champions, the project engaged with men and boys and women and girls in the wider community in the process of transforming harmful gender norms through a series of ongoing ‘community conversations’. These were six-week cycles of group discussions. Community Action Groups (CAGs) were also set up in each village, with members consisting of individuals with experience in or relevance to survivor support (e.g. health workers, survivors’ family members, etc.), and each CAG had at least one faith leader as a member. While gender champions were not required to be part of a CAG, in many cases they were. CAG members functioned as reference points for survivors in the community, to be accessed for support and referrals. CAG members were trained to share information in talks and discussion groups. All CAG members also received training in order to be able to provide basic psychosocial support to survivors of VAWG, and help rape survivors to access medical treatment, including post-exposure prophylaxis, at the nearest reference hospital in Rethy. By endline, 381 SGBV survivors had been supported, of which 61 had experienced sexual violence.

**Fig. 1:**
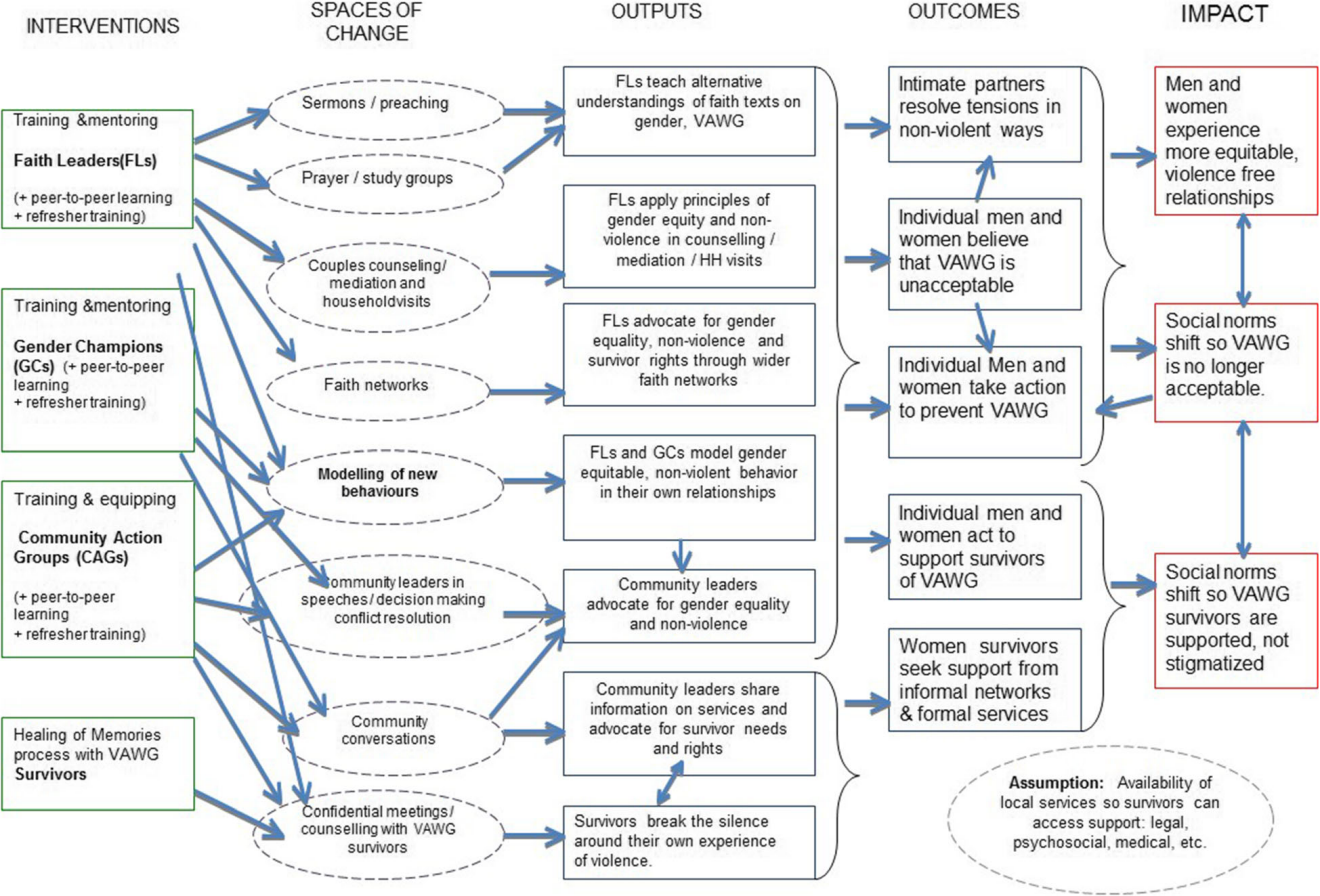
Project Theory of Change

The project’s Theory of Change (ToC) (see Fig. 1) is based on the understanding that belief systems and interpretations of faith texts can often support patriarchal social norms, and the assumption that faith leaders, as key local influencers, can be mobilised and equipped to become effective catalysts within their own communities, to address the underlying root causes of VAWG from a faith perspective. Thus it argues for the training and support of faith leaders, gender champions, CAGs, and a Healing of Memories process with survivors. This leads to a change in social norms so that VAWG becomes unacceptable and survivors are supported and not stigmatised, and men and women of the community are in more gender equitable, violence-free relationships.

The core of the Transforming Masculinities intervention comprises of the initial training of faith leaders and gender champions, community dialogue cycles of 6 weeks with a refresher training after the first cycle, and continuous monitoring and mentoring of the trained actors (faith leaders and gender champions). There is no established duration for the implementation of this approach, as the cycles can be repeated and adjusted to the context. For example, in the DRC some communities reached the 17th cycle of community dialogues, more refresher trainings were added, as well as specific trainings on particular topics (e.g. counselling). In the DRC the overall project started in April 2015 (with the first in-community activities in August 2015) and terminated in March 2018. The total length of implementation was 36 months (of which 29 months were direct work in the selected communities). For an overview of the intervention timeline, please see Table 1.

**Table 1:**
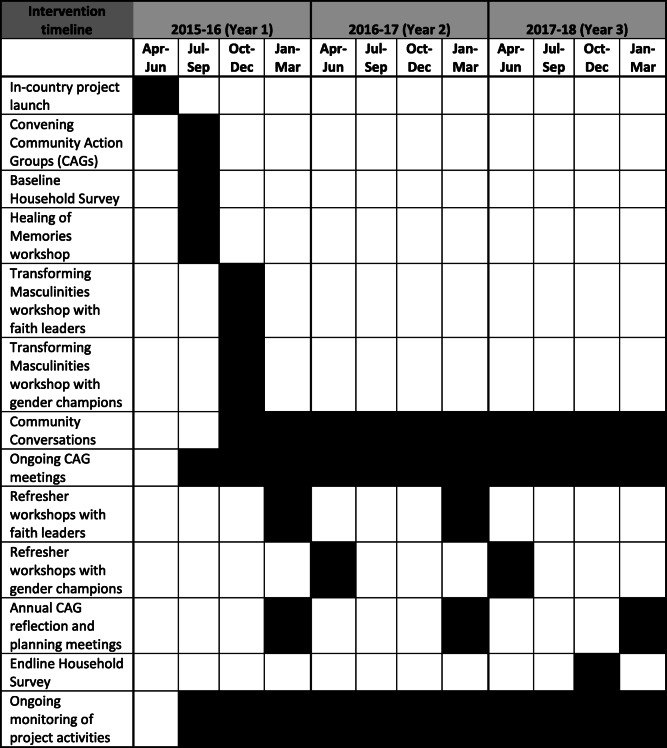
Intervention timeline

## Methods

### Setting

Research took the form of two community surveys, conducted before and after the intervention, and was conducted in three health areas (*aire de santé*) in Ituri Province, in the north-east of the DRC. The baseline survey was conducted in July 2015, while the endline survey was conducted at the end of November 2017. At both baseline and endline, data was collected from male and female members of randomly selected households in 15 villages (five per health area) in which the intervention was being implemented.

### Sample

At baseline, the aim of the sampling was to approach 800 households for interviews, with 400 male interviews and 400 female. The sample was divided equally between the three Health Areas and the five villages in each. Within the villages, households were selected for interview proportionate to the number of households in the village. The total number of households was divided by the number to be conducted to get the sampling interval and a transect walk was conducted with a random start (pen spun in the middle of the community) and every Nth household was approached for an interview. Within each household the household head or spouse was invited for interview and if they were not available other household members were selected to supplement the older and younger age groups of the sample. Interviews were conducted with one male or one female member per household. The sample was to be stratified according to gender (50:50 male: female) and age group. An age stratification guide was based on rural age distributions from the 2013 DHS survey. A total of 751 interviews are included in the baseline sample, with 387 female and 364 male respondents. This sample does not include 18 interviews that were conducted with respondents under the age of 18, which were removed from the baseline dataset.

At endline, there remained sufficient budget to increase the sample sizes, so the evaluation aimed to increase the sample by approximately 50% in order to improve the power of the sample as much as possible. In the end a total of 1198 interviews were included in the final sample (597 men and 601 women). This sample does not include 20 interviews that were conducted with respondents under the age of 18, which were removed from the endline dataset. The sample was again equally split between the three health areas and the aim was to conduct interviews with 50% male and 50% female respondents. Furthermore, at endline the opportunity was also taken to improve any bias in the sampling. At baseline, gender and age stratification was used as a non-probabilistic selection method that was easy to administer at low cost. Given some flexibility in the budgeting, it was decided that at endline this should be replaced by a birthday selection method, which maintains randomness yet is easy to administer and is not especially time consuming. Respondents were randomly selected at the household level after conducting transect walks as per baseline. In households where more than one eligible participant was available, the person with the most recent birthday was invited to participate in an interview.

### Administration

Interviews were conducted face-to-face by male and female enumerators and were administered using tablets loaded with the FormAgent Android app, with data uploaded directly to a server in real time if there was sufficient internet connection, or saved on the device where internet was poor. At both evaluation points, there were challenges recruiting a sufficient number of female enumerators to conduct sex-matched interviewing across the sample. Therefore, both male and female enumerators conducted interviews with male and female respondents. The enumerator selection process prioritised health workers on the basis that they would be qualified to discuss sensitive matters in an appropriate manner, so the team comprised practitioners from public health facilities and HIV projects. A section of the questionnaire including sensitive questions related to experience or perpetration of violence, was self-completed by participants. These measures have likely mitigated any potential issues with opposite-sex interviewing, as no significant interviewer effects related to the gender of enumerators were observed when analysing data related to experience or perpetration of violence. All questions in the self-administered section followed the same format. At the start of the section, enumerators walked respondents through the process using an example question (with no content). The enumerator would pose the question then hand the device to the respondent, who was instructed to tap the relevant icon, tap the ‘next’ button, and then return the device to the enumerator who would then read and pose the following question, and so on.

### Questionnaire

Baseline data collection took place in June and July 2015 and endline data collection took place in November and December 2017, with approximately 29 months between evaluation points.

The survey questionnaire was designed in English. At baseline it was translated into French and then administered by enumerators in local languages (mainly Kilendu) through on-the-spot translation. At baseline, enumerators practiced administering the survey in local languages during training and discussed how certain questions should be worded in local languages. As a further measure to ensure consistency of questioning, the endline questionnaire was translated from English into French and then subsequently translated into Kilendu. Enumerators used both French and Kilendu versions in the field depending on language requirements of participants, and conducted on-the-spot translation where respondents spoke a different language. In practice, some enumerators were not familiar with written Kilendu, preferring to read in French, which gives confidence in the baseline approach.

The baseline and endline questionnaire included a range of questions to obtain demographic information about respondents, including gender, age, educational level, current marital and relationship status, employment and economic conditions, and religious affiliation and involvement. Age was measured through a continuous variable and then converted into a categorical variable. Educational level was measured by asking the respondent’s highest level of school attended. The baseline survey captured respondents who were married or co-habiting, while the endline survey expanded this scope to include those in a boyfriend/girlfriend relationship i.e. they had a regular sexual partner.

Poverty, assessed in terms of food security, was measured through a number of variables. At baseline and endline, a question was asked about the number of meals respondents’ households generally had in 1 day. In the endline survey three additional questions were included to measure the past month frequency of: (1) no household access to food of any kind, (2) any household member going to sleep at night without eating and (3) any household member going for a whole day without food. For these three questions, possible response options were never, rarely, sometimes or often. A food insecurity score was created by summing the values of these three variables (score range 0–9).

Respondents were asked about their religious affiliation, importance of faith, and attendance at religious institutions. Respondents were also asked about their degree of faith engagement. At baseline a three level ‘faith engagement’ variable was developed grouping (1) no engagement at all or no religion, (2) simply attending services, and (3) all responses pertaining to taking part in services and engagement in decision-making or leadership. This was done after testing religion, attendance and faith engagement variables to determine which was more predictive of attitudes towards violence and gender, and finding that the faith engagement variable was the strongest predictor. The same faith engagement variable was developed at endline.

The baseline and endline surveys include a number of items to test social norms, including attitudes and beliefs related to gender equality, gender roles and masculinities, and attitudes towards gender, sex and violence. Four composite scales were created by grouping variables together, including: a gender attitudes scale, a masculinity attitudes scale, a rape myth scale and a rape stigma scale. For all four composite scales, questions were measured on a five-point Likert scale (strongly disagree to strongly agree), with corresponding values ranging from 1 to 5. The values for negatively worded items were reversed, and item values summed to create scores. Full details of variables included in each scale are presented in Table 2, and a description of each composite scale is outlined below. Inclusion of scale items was determined by analysing internal consistency using Cronbach’s alpha, with 0.70 or more (or close to this) used as the indicator of good internal consistency.

**Table 2:** Questionnaire items used to construct outcomes

	Indicator	Respondents	Items in composite indices	Expected direction of change
Gender attitudes	Mean score: gender attitudes	Male, female	(1) A good woman obeys her husband even if she doesn’t agree, (2) changing nappies, giving a bath and feeding children is the mother’s responsibility, (3) a woman’s primary role is to take care and cook for her family, (4) a man should have the final word about decisions in his home, (5) when married, a woman has no right or control over her body according to scriptures, (6) when a man has paid bride price his wife becomes his property, (7) men are superior to women	Increase (higher scores indicate more equitable attitudes)
Attitudes towards masculinities	Mean score: attitudes towards masculinities	Male, female	(1) It is important for a man to demonstrate that he is the head of the house, even using violence, (2) to be a man, you need to be tough, (3) it is manly to defend the honour of your family even by using force, (4) it is manly for a man to beat his wife	Increase (higher scores indicate more equitable attitudes)
Rape myths	Mean score: beliefs in rape myths	Male, female	(1) When a woman is raped, she usually did something careless to put herself in that situation, (2) in some rape incidents the victims actually want it to happen, (3) if a woman doesn’t physically fight back, you can’t really say it was rape, (4) in any rape incident one would have to question if the victim had a bad character, (5) God condemns rape	Increase (higher scores indicate less agreement with rape myths)
Rape stigma	Mean scores: agreement with rape stigma	Male, female	(1) A man is justified in rejecting his wife if she has been raped, (2) A raped woman’s family members should have nothing to do with her, (3) A young man should not marry a young woman who has been raped	Increase (higher scores indicate less agreement with rape stigma)
Emotional IPV	% of respondents who report at least once instance of violence in the past 12 months	Female, male	Female: In the past 12 months, how many times has your husband, partner or boyfriend done the following things to you – (1) Belittled or humiliated you in front of other people, (2) threatened to hurt you or someone you care about. Male: In the past 12 months, how many times have you done the following things to your wife, partner or girlfriend – (1) Belittled or humiliated her in front of other people, (2) threatened to hurt her or someone she cares about.	Decrease
Physical IPV	% of respondents who report at least once instance of violence in the past 12 months	Female, male	Female: In the past 12 months, how many times has your husband, partner or boyfriend done the following things to you – (1) Pushed or shoved you, (2) slapped you or thrown something at you which could hurt you, (3) hit you with his fist or with something else that could hurt you, (4) kicked you, dragged you, beat you, strangled or burned you, (5) threatened you or attacked you with a gun, knife or other weapon. Male: In the past 12 months, how many times have you done the following things to your wife, partner or girlfriend – (1) Pushed or shoved her, (2) slapped her or thrown something at her which could hurt her, (3) hit her with your fist or with something else that could hurt her, (4) kicked her, dragged her, beat her, strangled or burned her, (5) threatened her or attacked her with a gun, knife or other weapon.	Decrease
Sexual IPV	% of respondents who report at least once instance of violence in the past 12 months	Female, male	Female: In the past 12 months: (1) How often has he physically forced you to have sexual intercourse when you did not want to, (2) how many times have you had sex with him because you were frightened he would become violent, (3) how many times did he force you to do sexual things which you didn’t want to do. Male: In the past 12 months: (1) How often have you physically forced her to have sexual intercourse when she did not want to, (2) how many times have you used threats or intimidation to make her have sex with you when she didn’t want to, (3) how many times did you force her to perform sexual things which she didn’t want to do.	Decrease

The baseline and endline questionnaires included 11 items about attitudes and beliefs related to gender equality, gender roles, household decision making and gender norms in religious texts and teachings. These items were adapted from a variety of sources including: the World Health Organisation (WHO) Multi-Country Study on Women’s Health and Life Events [30], the International Men and Gender Equality Survey (IMAGES) [31], the DHS [10] and the Gender Equitable Men (GEM) Scale [32]. Additional questions aligned with gender and religious norms were adapted by Tearfund. When first producing a gender attitudes composite scale, there were problems with internal scale consistency when four particular items were included.^2^ The four variables that impacted negatively on the internal consistency of the scale were subsequently dropped, leaving seven variables (see Table 2). The score range for the gender attitudes scale is 7 to 35, with higher scores indicating more equitable gender attitudes. For male respondents Cronbach’s alpha was 0.69 at baseline and 0.75 at endline, and for female respondents Cronbach’s alpha was 0.69 at baseline and 0.76 at endline.

The baseline and endline questionnaires included five items about attitudes and beliefs related to masculinities, developed by Tearfund to support their work on masculinities. After testing for the internal consistency of a masculinities composite scale, one item was dropped to improve internal consistency, leaving a composite scale comprising four variables (see Table 2). The score range for the attitudes towards masculinities scale is 4 to 20, with higher scores indicating more gender equitable attitudes related to masculinity. For both male and female respondents, internal scale consistency was high: Cronbach’s alpha was 0.86 at baseline and 0.87 at endline for men, and 0.80 at baseline and 0.87 at endline for women.

Five items related to beliefs in common rape myths were included in the baseline and endline survey and all five items were included in the corresponding composite scale (see Table 2). The score range for the rape myths scale is 5 to 25 with higher scores indicating less agreement with rape myths. Cronbach’s alpha was 0.77 at baseline and 0.75 at endline for men, and 0.73 at baseline and 0.76 at endline for women. Five items were also included to measure agreement with statements that stigmatise survivors of rape. After testing for the internal consistency of a rape stigma composite scale, two items were dropped leaving a composite scale comprising three items. The score range for the attitudes towards rape stigma scale is 3 to 15, with higher scores indicating less agreement with stigmatising attitudes and thus more positive attitudes. Cronbach’s alpha was 0.71 at baseline and 0.72 at endline for men, and 0.67 at baseline and 0.79 at endline for women.

A range of survey questions were included to measure men’s and women’s attitudes towards IPV. Three survey items were included to measure responses to tolerating or interfering in IPV: (1) If a man mistreats his wife, others outside of the family should intervene, (2) A woman should tolerate violence to keep her family together, and (3) A man using violence against his wife is a private matter that shouldn’t be discussed outside the couple. All three questions were measured on a five-point Likert scale, with response options later collapsed into three categories (agreement, neither, or disagreement). A range of questions were also included to measure respondents’ perceptions related to physical and sexual IPV and scenarios for justification of physical and sexual IPV. Two items measuring perceptions of IPV (There are times when a woman deserves to be beaten, and A man is entitled to sex from his partner even if she doesn’t feel like it) were measured on a five-point Likert scale, with response options later collapsed into three categories (agreement, neither, or disagreement). Attitudes towards a husband’s justification for beating his wife were measured through agreement or disagreement with eight scenarios (see Table 4). Responses were recorded nominally (yes/no). Attitudes towards a woman’s ability to refuse sex were measured through agreement or disagreement with four scenarios in which a woman can refuse sex: if she doesn’t want to, if he is drunk, if she is sick and he mistreats her. Responses were recorded nominally (yes/no).

Women’s experience or men’s perpetration of past 12 month emotional, physical or sexual intimate partner violence (IPV) were measured through a range of items asked of all respondents who were married or living with a partner, or who had had a relationship in the past 12 months (Table 2). Women’s experience of past 12 month emotional IPV was measured through two items derived from the DHS domestic violence module. Women’s experience of past 12 month physical or sexual IPV was measured through items obtained from the WHO Multi-Country Study on Women’s Health and Domestic Violence [30]. Men’s perpetration of IPV was measured through the same items used for women but worded in the active voice, as conducted in the United Nations Multi-Country Cross-Sectional Study on Men and Violence in Asia and the Pacific [33]. For each type of IPV measure (emotional, physical or sexual), items were recorded as never, once, a few times or many times and experience or perpetration of IPV was coded if respondents reported any act on one or more occasions.

The questionnaire also included questions about acts of sexual violence from, or perpetrated against, a person who was not an intimate partner. There was a small difference in question wording between the baseline and endline questionnaires for one item asked of women. At baseline, women were asked: ‘In the past 12 months, how many times has someone other than your partner, husband or boyfriend forced you to have sex’ (never, once, a few times or many times)? At endline, the question was worded: ‘In the past 12 months, how many times has someone other than your partner, husband or boyfriend forced you to have sex or do something sexual you didn’t want to do’? However, there is little reason to believe that the difference in wording would account for any change in non-partner sexual violence (NPSV), given that it would be unusual for women to report having to do something sexual they did not want to without forced sex being involved. Women were also asked a series of questions to determine if the perpetrator was known or unknown to her, who it was (family member, soldier/armed militia, other) and whether there were multiple perpetrators. At endline only, men were asked ‘In the past 12 months, how many times have you forced any other woman to have sex with you’.

It should be noted that no other organisation was implementing any intervention in this geographical location at the same time as this project. A number of survey items were included in the endline survey to record exposure to the intervention according to three key groups: those who participated in *counselling* (if a respondent attended either couples counselling or teaching or counselling on SGBV in the past 12 months); those who participated in a talk or discussion group (if the respondent heard a talk or attended a discussion group, sermon or public talk at a religious institution or elsewhere in the past 2 years); and those who participated as a programme actor (respondents who said they were a Faith Leader, Gender Champion, or a member of a Community Action Group). For counselling, the measurement of past 12 month exposure was selected due to counselling being implemented in the final year of the project after selected faith leaders received counselling training. Counselling activities had always been led at various degrees by the faith leaders as part of their existing activities with couples and individuals in their community and faith groups. This was thus not an additional activity within the intervention, but the faith leaders were expected to lead this activity in light of the learning and mentoring received. However, an additional training on counselling –not originally part of the intervention- was provided by a consultant to ensure consistency and strengthen the faith leaders’ skills in this area. In contrast, exposure to talks or discussion groups was measured for the past 2 years given that this activity had been implemented (by gender champions as part of community dialogues) since the first year of project implementation.

### Data analysis

Data collected at both evaluation points was downloaded and compiled into a single dataset identified by data collection wave. Data was analysed using SPSS and Stata 13. Categorical variables were summarised as percentages, with Pearson’s chi-square tests used to test for statistical significance between baseline and endline samples. Ordinal variables for Likert scales are summarised as percentages with Mann Whitney U tests to conduct significance tests. Means are reported for continuous/scale data, with Mann Whitney U tests conducted to test for significance between baseline and endline samples where independent variables are nominal with two groups, and Kruskal Wallis tests conducted where independent variables have more than two groups (e.g. in the case of the three-level variable for faith engagement). Non-parametric tests (Mann Whitney U and Kruskal Wallis) were used as they tend to be more robust when working with ordinal data as they are based on fewer assumptions, especially assumptions of normal distribution given that attitudinal measures with the kind of five point Likert scales used in the survey are often highly skewed.

Propensity score matching was conducted to identify whether differences between baseline and endline reports of IPV could be explained by social and demographic differences between the samples. The matching was conducted in two ways, first on age and education and secondly on age, education and number of meals consumed per day. In each case all reductions in IPV were statistically significant at *p* <  0.0001, except women’s experience of physical IPV in the second model where *p* = 0.002. Thus, we found no evidence that reductions in IPV were due to structural differences in the sample.

### Ethics and safety

Ethics approval for this research was granted by the ethics committee of the Université Libre Pays des Grands Lacs in Goma, DRC. Permission was also granted by the Provincial Health Division of Ituri (Ministry of Public Health), as well as agreed in advance with community leaders in each of the targeted villages. Informed consent was required for each person interviewed, and sensitive questions on experience or perpetration of violence were all self-completed by the participants, so that enumerators did not know the responses to these questions.

Enumerators were trained on ethical principles (respect, confidentiality, consent, safety, Do No Harm, referrals) and were provided with leaflets to share with participants, with phone numbers of project staff to contact in case any questions or issues arose during the survey. These leaflets were also shared where participants requested support through referrals, particularly survivors of violence requesting psychosocial support. Details of local clinics were also made available, so that the enumerators had the necessary information available.

At both baseline and endline evaluation points, a key eligibility criteria was that respondents should be aged 18 years or older. At baseline 18 respondents under the age of 18 years were sampled and at endline 20 respondents under the age of 18 were sampled. Enumerators erroneously judged that these respondents could be interviewed, as they had primary responsibility for a household (for example, two 15-year olds were considered adults as they were both married with children). These cases were removed from the final datasets in order to comply with the ethics protocols of the study.

## Results

### Demographics

The mean age of the baseline sample was 30.5 years with a range of between 18 and 75 years, and the endline sample was older with a mean age of 35.6 years and a range of between 18 and 87 years. The mean age of male respondents was higher than female respondents at both baseline (male: 32, female: 29.1) and endline (male: 36.8, female: 34.4). Table 3 shows that the endline sample had fewer respondents aged 18 to 24, and more over 50 year olds, compared to the baseline sample, with smaller differences between the baseline and endline samples for other age groups. Most respondents were in some form of relationship at the time of both surveys.^3^ The proportion in some kind of intimate partner relationship was similar in both samples, 75.2% in the baseline sample and 80.4% in the endline sample, although the proportion of respondents who were married or cohabiting was greater at endline. The distribution of level of education attained by respondents from the two surveys was similar. The proportion of respondents with no education was lower in the endline sample, and the overall education status of the endline sample was slightly higher. More respondents in the endline sample reported being food secure compared to the baseline sample. More food security at endline may have been a consequence of differences in the seasonal timing of the surveys, as the endline survey was conducted at harvest time.

**Table 3:** Baseline and endline demographic data

	Baseline		Endline		
	n	%	n	%	***P*** value
**Age**					< 0.001
18–24	284	38.1	320	26.9	
25–34	229	30.7	327	27.4	
35–49	184	24.7	344	28.9	
50 +	49	6.6	201	16.9	
**Marital status**					< 0.001
Married	–	–	668	55.9	
Married or cohabiting	359	48.1	–	–	
Formerly married but currently unmarried	215	28.8	340	28.4	
Never married	172	23.1	188	15.7	
**Relationship status**					< 0.001
Married or cohabiting	361	48.1	729	61	
Currently has regular sexual partner	170	22.6	57	4.8	
Partner in the last 12 months but currently has no sexual partner	34	4.5	174	14.6	
No relationship in last 12 months	186	24.8	235	19.7	
**Education level**					< 0.001
None	207	27.6	272	22.7	
Incomplete primary	253	33.7	386	32.2	
Complete primary	102	13.6	202	16.9	
Incomplete secondary	103	13.7	237	19.8	
Complete secondary	66	8.8	87	7.3	
Post-secondary education	20	2.7	14	1.2	
**Meals per day**					< 0.001
3 or more meals	173	23	375	31.8	
2 meals	478	63.7	699	59.3	
1 meal	100	13.3	104	8.8	

### Social norms and attitudes

The baseline and endline results for four composite scales related to social norms are listed in Table 4. Both male and female respondents had significantly more equitable gender attitudes at endline, with significant improvements observed in all survey items related to gender equality, attitudes towards power in relationships, and gendered norms around household decision making (data not shown). There were no significant differences between baseline and endline in mean masculinities attitudes scores for either men or women, although a large majority of respondents in both samples rejected attitudes linking use of violence to masculine identity. Although men reported significantly less agreement with rape myths at endline, the same trend was not observed for women. Nevertheless, both men and women had significantly higher stigma scores at endline, indicating a reduction in attitudes that stigmatise rape survivors.

**Table 4:** Baseline and endline mean scores and frequencies for social norms and attitudes associated with gender and violence against women, disaggregated by gender of respondent

	Male respondents			Female respondents		
	Baseline %/Mean	Endline %/Mean	*P* value	Baseline %/Mean	Endline %/Mean	*P* value
**Social norms composite scales (mean)**						
Gender attitudes scale	15.7	18.6	< 0.001	16.4	18	< 0.001
Masculinities attitudes scale	14.7	14.9	0.159	15.4	15.2	0.853
Rape myths scale	16	16.7	0.001	16.8	16.9	0.746
Stigma scale	9.9	10.6	< 0.001	10.2	10.6	0.002
**Agreement with statements associated with justification for physical IPV (%)**						
There are times when a woman deserves to be beaten	51.1	28.2	< 0.001	41.6	23.7	< 0.001
A husband is justified in beating his wife in the following situations:						
If she goes out without telling him	31.7	24.1	0.010	26.7	30.1	0.259
If she neglects the children	31.7	20.8	< 0.001	26.2	27	0.785
If she argues with him	35.8	26.2	0.002	34.1	32.3	0.574
If she refuses to have sex with him	41.8	23.4	< 0.001	34.9	31.9	0.334
If she burns the food	14	11.9	0.344	15.5	15.8	0.903
If he is not satisfied with the way she does the housework	24.6	15.6	0.001	19.6	21.1	0.593
If she disobeys him	56.1	31.1	< 0.001	52.4	38	< 0.001
If he finds out that she has been unfaithful	64	47.2	< 0.001	64.5	61.2	0.302
**Agreement with statements associated with justification for sexual IPV**						
A man is entitled to sex from his partner even if she doesn’t feel like it	76	39.9	< 0.001	67.6	45	< 0.001
A woman is able to refuse sex in the following situations:						
If she doesn’t want to	34.5	65.5	< 0.001	40.4	59.8	< 0.001
If he is drunk	34	64.3	< 0.001	39.6	63.6	< 0.001
If she is sick	59.8	78.1	< 0.001	59.1	74.6	< 0.001
If he mistreats her	42.7	68.5	< 0.001	45.3	68.5	< 0.001
**Agreement with statements associated with response to IPV (%)**						
A woman should tolerate violence to keep her family together	62.7	36.2	< 0.001	47.4	35.8	< 0.001
A man using violence against his wife is a private matter that shouldn’t be discussed outside the couple	53.9	32.1	< 0.001	51.4	32	< 0.001
If a man mistreats his wife, others outside of the family should intervene	90.6	86.9	0.075	91.2	80.2	< 0.001

At baseline 51.1% of men and 41.6% of women agreed that there are times when a woman deserves to be beaten (Table 4). However, the proportion of both male and female respondents agreeing with this statement almost halved at endline, indicating a strongly significant reduction in violence-supportive attitudes. The proportion of male respondents agreeing that physical IPV is justified in eight different scenarios was significantly lower at endline for all types of scenarios except if a woman burnt the food, which was already poorly supported at baseline. The same pattern was not observed for female respondents, with a significant reduction in violence-supportive attitudes at endline only found in response to whether a husband is justified in beating his wife if she disobeys him. The endline findings were more uniform for male and female respondents in relation to sexual IPV. Significantly fewer male and female respondents at endline reported agreeing that a man is entitled to sex from his partner even if she doesn’t feel like it. Furthermore, significantly more male and female respondents at endline agreed that a woman could refuse sex in any of four scenarios.

Men in the baseline sample had a higher expectation than women that women should tolerate violence as part of keeping the family together (Table 4). However, among respondents in the endline sample, the proportions of men and women agreeing with this statement have reduced significantly and the gender gap has been eliminated. Support for discussing violence has increased significantly among both men and women. However, agreement that people should intervene if a man mistreats his wife is lower at endline among both male and female respondents, although the difference is only sigificant for female respondents.

### Experience and perpetration of IPV and non-partner sexual violence

Among those women who reported being in a relationship currently or in the past 12 months, there has been a significant decline at endline in reports of emotional, physical and sexual IPV, with reports of any kind of IPV more than halving between both evaluation points (Table 5). The same trend is observed for reports of IPV perpetration by men in a relationship currently or in the past 12 months. Endline prevalence of IPV perpetration reduced significantly to approximately a third of the baseline prevalence of violence perpetration for all three types of IPV. At both baseline and endline, reported rates of IPV experienced by women are consistent with rates of perpetration reported by men.

**Table 5:** IPV in the last 12 months (male perpetration, female experience)

	Male			Female		
	Baseline	Endline	*p* value	Baseline	Endline	*p* value
Emotional IPV	51%	13.7%	< 0.001	50%	18.4%	< 0.001
Physical IPV	35.1%	12%	< 0.001	30.3%	16.6%	< 0.001
Sexual IPV	31.4%	8.5%	< 0.001	36.8%	15.1%	< 0.001
Any IPV	68.2%	23.3%	< 0.001	68%	29.3%	< 0.001

There was a large reduction between the baseline and endline surveys in the proportion of women who reported having experienced NPSV in the past 12 months (20.7% at baseline compared with 3.7% at endline). In relation to the type of perpetrator, although the number of cases of NPSV reported was small at endline (*n* = 22) compared with baseline (*n* = 64), there appears to have been a reduction at endline in the proportion of family members as perpetrators of NPSV (4.6% compared with 18.8% at baseline), with a corresponding increase in other known perpetrators at endline (86.4% compared with 67.2% at baseline). At both evaluation points, the proportion of women reporting NPSV who named militia as the perpetrator was small (6.3% at baseline and 9.1% at endline).

### The role of faith engagement

Adherence to a religion was high (baseline 95.5% and endline 95.9%). The majority of respondents were Christian, and the proportion of Christians was slightly higher among the endline sample (Table 6). Most respondents who identified as belonging to a religion in the baseline sample considered that their faith was important or very important to them (83.6%). However, importance of faith is more prominent in the endline sample, with the proportion of respondents reporting their faith to be important or very important rising to 95.5% (*p* <  0.001). The proportion of respondents who did not attend religious institutions did not differ between baseline and endline; however, the proportion of respondents who reported regularly attending services or prayers was almost twice as high at endline. The proportion of respondents not engaged at all with a religious institution was slightly higher in the endline than baseline sample; however, respondents in the endline sample reported more active engagement overall. Approximately half (51.8%) of baseline respondents who said they were ‘not engaged at all’ did attend their religious institution in some capacity and this proportion was 62.2% in the endline sample. These represent what appears to be a passively engaged congregation.

**Table 6:** Religious and faith participation and engagement

	Baseline (%)	Endline (%)	***p*** value
**Religion**			0.002
Christian	76.3	81.2	
Muslim	14.4	11.4	
Traditional religion	0.9	1.8	
Other (incl. Kimbanguist)	3.9	1.6	
None	4.5	4.1	
**Importance of faith**			< 0.001
Don’t know / no opinion	13.3	0	
Not at all important	0.8	1.4	
Not important	2.2	3.2	
Important	62	77.1	
Very important	21.6	18.4	
**Attendance at religious institution**			< 0.001
Doesn’t attend	17.3	15.2	
Occasionally attends services/prayers	31.4	22.6	
Occasionally attends other activities	11.2	11.7	
Regularly attends services/prayers	24	42.8	
Regularly attends both services/prayers and other activities	16.1	7.8	
**Engagement with religious institution**			< 0.001
Not engaged at all	34.8	39	
Just attends services	22.4	13.9	
Takes part in the services	34	36.8	
Takes part in decision making	4.9	6.8	
Involved in leadership	3.9	3.6	

When disaggregating baseline social norms composite scale scores by faith engagement, those who were faith engaged (attending services or taking part in them) had significantly more gender equitable attitudes, more gender equitable attitudes related to masculinities, and less agreement with rape myths or rape stigma than respondents with no religion (Table 7). Those taking part in services reported the most positive social norms overall. At endline, the same pattern in results was observed only for gender equitable attitudes related to masculinities and less agreement with rape myths. When comparing baseline and endline scores, gender attitudes, rape myths and rape stigma scores have increased for all three types of faith engagement, indicating more equitable gender attitudes and less agreement with rape myths and rape stigma across the board. However, masculinities scores have stayed the same except for those respondents who take part in services, among whom we see an increase in gender equitable attitudes related to masculinities.

**Table 7:** Baseline and endline results for social norms, attitudes and experience and perpetration of IPV, disaggregated by faith engagement

	Baseline (mean/%)				Endline (mean/%)			
	No religion	Attends services	Takes part	***p*** value	No religion	Attends services	Takes part	***p*** value
**Social norms composite scales (mean)**								
Gender attitudes scale	15.6	15.3	16.8	< 0.001	18.2	18	18.5	0.162
Masculinities attitudes scale	14.6	15	15.5	< 0.001	14.5	15.7	15.3	< 0.001
Rape myths scale	15.8	17.3	16.5	< 0.001	16.2	17.2	17.2	< 0.001
Stigma scale	9.7	10	10.4	< 0.001	10.5	10.7	10.6	0.615
**Agreement with statements associated with justification for and response to IPV (%)**								
A woman should tolerate violence to keep her family together	49.5	44.7	65.4	< 0.001	39.7	31.5	33.7	0.019
A man using violence against his wife is a private matter that shouldn’t be discussed outside the couple	52.7	54.4	51.6	0.525	36.6	28.9	28.7	0.006
If a man mistreats his wife, others outside of the family should intervene	89.4	86.8	94.8	0.004	75.8	89.9	88.7	< 0.001
There are times when a woman deserves to be beaten	46.6	56.3	40.9	0.002	29.2	23.3	23.6	0.027
A man is entitled to sex from his partner even if she doesn’t feel like it	73.1	79.4	66.5	0.001	52	43.4	33.3	< 0.001
**Past 12 month experience of IPV (female respondents) (%)**								
Emotional IPV	52.9	35.4	57.6	0.019	18.5	23.8	16	0.319
Physical IPV	37.1	24.6	29.4	0.275	16.3	17.5	16.5	0.971
Sexual IPV	48.6	41.5	23.9	0.003	17.4	16.3	12.4	0.373
Any IPV	78.6	55.4	68.5	0.015	29	37.5	26.3	0.178
**Past 12 month perpetration of IPV (male respondents) (%)**								
Emotional IPV	69.1	35.2	44.9	< 0.001	12.5	6.4	16.6	0.088
Physical IPV	47.6	38.9	23.4	0.002	12.5	7.9	12.7	0.570
Sexual IPV	39.3	38.9	21.5	0.013	10.7	6.4	6.6	0.222
Any IPV	81	61.1	61.7	0.008	24	17.5	23.6	0.529

Among respondents in the baseline sample, it was those who actively engaged with their faith that had the highest agreement that a woman should tolerate violence (Table 7). However, by the time of the endline survey, this agreement reduced across all faith engagement categories, but especially among the actively engaged group where a 50% reduction in agreement with women’s tolerance of IPV was observed. In the baseline sample, it was the actively faith engaged group that agreed almost universally that people should intervene in cases of violence. Endline support for intervening declined among respondents with no religion and those who took part in services and increased slightly for those attending services. A smaller proportion of actively faith-engaged respondents at baseline than respondents with no religion or those who attended services agreed that a man using violence against his wife is a private matter that should not be discussed outside the family. At endline, the proportion of respondents agreeing that violence should not be discussed was smaller for all faith categories, although agreement was lowest for both of the faith engaged groups. At baseline, respondent agreement that there are times when a woman deserves to be beaten was lower among those who took part in religious services and those who only attended than respondents with no religion. At endline, agreement was lower for all three groups, with agreement remaining lowest for those who attended services and those taking part, when compared with respondents with no religion. The same pattern is observed for agreement with the statement that a man is entitled to sex from his partner even if she does not feel like it. The proportion of respondents agreeing with the statement was smaller at endline for those in all three faith categories, with the lowest agreement evident for those respondents who take part in faith engagement services.

Baseline reports of past 12 month perpetration of IPV by men showed that faith engaged men (attending services or taking part in them) perpetrated less IPV. However, this same effect was not seen at endline, although IPV perpetration by all three groups had very substantially decreased. Baseline reports of past 12 month experience of IPV showed slightly less IPV experience by faith engaged women (attending services or taking part in them). Once again, this same effect is not seen at endline, although IPV experience by all three groups had very greatly decreased.

### Exposure to intervention activities

At endline respondents indicated whether they were directly involved in the intervention as a ‘programme actor’, as a faith leader (17%), a gender champion (9.3%), or a member of a community action group (CAG) (12.9%). Gender champions and CAG members were well balanced in terms of gender, but a higher proportion of faith leaders were male (58.3%, *p* = 0.008). This is the largest group of actors, making the overall group of actors male biased (*p* = 0.01). A very large proportion of respondents reported having participated in counselling (68.8%) or talks/discussions (82.9%). There was not a significant difference between the proportion of endline male (82.2%) and female (83.5%) respondents who had attended public talks or discussions or counselling (men 70.6% and women 67%). There was overlap between these activities as 80.3% of those who attended a public talk or discussion also attended counselling.

The mean endline social norms composite scale scores were significantly higher among respondents who attended counselling or a public talk/discussion (Table 8), indicating more equitable social norms among those exposed to intervention activities, except in the measure of rape myths where there was no difference found between those who had or had not attended counselling. Similarly, for statements associated with women tolerating IPV, IPV not being discussed outside of the couple, outside intervention in cases of IPV, and support for physical and sexual IPV, those respondents who attended counselling or public talks/discussions had significantly less agreement with the statements than those respondents who had not participated in counselling or public talks/discussions on almost all measures. There are two exceptions. One is with outside family intervention in cases of IPV between those respondents who had or had not participated in counselling or public talks/discussion. The other exception is with violence being a private matter between respondents who had or had not participated in counselling (although the effect is in the right direction and is almost significant). Although endline attitudes and social norms were more gender equitable among those participating in counselling or public talks or discussions, there was no difference in reports of past 12 month experience (by women) or perpetration (by men) of IPV according to participation in intervention activities.

**Table 8:** Endline results for social norms, attitudes and experience and perpatration of IPV, disaggregated by exposure to intervention activities

	Attended counselling?			Attended public talk or discussion?		
	Yes (%/mean)	No (%/mean)	*p* value	Yes (%/mean)	No (%/mean)	*p* value
**Social norms composite scales (mean)**						
Gender attitudes scale	18.6	17.5	< 0.001	18.7	16.4	< 0.001
Masculinities attitudes scale	15.2	14.6	0.011	15.3	13.9	< 0.001
Rape myths scale	16.9	16.6	0.335	17	15.7	< 0.001
Stigma scale	10.9	9.9	< 0.001	10.8	9.5	< 0.001
**Agreement with statements associated with justification for and response to IPV (%)**						
A woman should tolerate violence to keep her family together	34.1	40.6	0.027	34.4	43.4	0.007
A man using violence against his wife is a private matter that shouldn’t be discussed outside the couple	30.5	35.8	0.064	29	46.8	< 0.001
If a man mistreats his wife, others outside of the family should intervene	82.4	86.3	0.090	83.8	82.4	0.886
There are times when a woman deserves to be beaten	23.2	31.8	0.003	22.8	40.5	< 0.001
A man is entitled to sex from his partner even if she doesn’t feel like it	37.6	53.1	< 0.001	37.9	64.4	< 0.001
**Past 12 month experience of IPV (female respondents)**						
Emotional IPV	17.6	20	0.528	18.2	19.2	0.827
Physical IPV	16.3	17.3	0.780	15.8	20.3	0.337
Sexual IPV	15.6	14.1	0.663	15.1	15.2	0.977
Any IPV	28	32.1	0.367	28.6	32.9	0.440
**Past 12 month perpetration of IPV (male respondents)**						
Emotional IPV	13.5	14.1	0.872	13.7	13.6	0.988
Physical IPV	13.3	8.5	0.130	12.8	8	0.203
Sexual IPV	9.1	7	0.451	9.1	4.6	0.156
Any IPV	24.5	20.4	0.329	24.2	18.2	0.222

## Discussion

We have shown that at endline there were significantly more equitable gender attitudes and less tolerance for IPV and a significant decline in all aspects of IPV in the communities who experienced the intervention. Although we have no comparison group, we have shown that there were very high levels of exposure to the intervention in the communities and that improvements in attitudes towards gender and the use of violence were more pronounced among those who had been involved in intervention activities. All of this is in keeping with the theory of change and the expectation that attitude change would precede behaviour change and suggests that the differences between endline and baseline may be attributable to the intervention. We noted that the experience and perpetration of IPV reported at endline did not track with exposure to the intervention, but it is plausible that in a context where social norm change was sought, the impact of the intervention on those exposed could have had an impact on the behaviour of the unexposed. This is in line with the ToC, which hypothesises that a shift in social norms amongst the exposed can result in a shift in the behaviour amongst the broader (unexposed) community.

We find that the samples used to study the programme had some differences despite both baseline and endline samples having been randomly drawn from the same three districts using a very similar sampling method. The endline sample were older, a little more educated, and had more food security than baseline respondents. However, very importantly, propensity score matching tested whether the differences in baseline and endline IPV prevalence were attributable to these sample differences and the conclusion was that demographic differences between the two samples were not responsible for changes we see in experience of IPV.

The programme ToC was predicated on the reach and role of religious institutions in insecure and remote rural communities in Sub-Saharan Africa. It proposed that social norms associated with VAWG, and with support for survivors, could be changed by supporting faith institutions to actively engage and advocate for an ending VAWG agenda. Training, mentoring and equipping of faith leaders, gender champions, and Community Action Groups would lead to social norms that condemn VAWG and support survivors, and more equitable, violence-free relationships at community level. Referring to the ToC, we find that the programme has been successful in affirming several of the key assumptions made in the ToC.

We found a *significant decline in all aspects of IPV at endline in comparison to baseline*, a result supported by the fact that reported rates of experiencing IPV is closely aligned with reported perpetration rates. Women’s experience of any form IPV decreased from 68 to 29.3%. Impact is seen not only in terms of intimate relationships, for non-partner sexual violence (NPSV) also decreased significantly. Women reporting experiencing NPSV in the past 12 months reduced from 20.7% to 3.7%, With NSPV perpetration by family members decreasing from 18.8 to 4.6%%. Considering that the WHO Multi-country Study on Women’s Health and Domestic Violence against Women [30] estimated that between 15 to 71% of ever-partnered women have experienced physical or sexual violence (with estimates from most sites ranging between 30 and 60%), and between 20 and 75% have experienced emotional abuse, the reduction in IPV in the 15 intervention communities is particularly significant.

While the analysis showed that reduction in IPV experience (by women) and perpetration (by men) at endline does not directly relate to level of faith engagement, this does not necessarily mean that faith engagement is not related to reduction in violence. It is possible that altered behaviours with less use of violence among the intervention exposed impacted across the community and resulted in a lesser experience and perpetration of IPV among everybody, and not just those engaged with a faith group. This was one of the core assumptions of the intervention and affirmed when reflecting on attitude change across the communities (see below).

It should be noted that the prevalence of IPV compared to NPSV (both at baseline and endline) highlights that, contrary to popular global perceptions about VAWG in the DRC, VAWG perpetrators are much more often intimate partners than unknown soldiers or militia members. This calls for a change in the global narrative around conflict-related VAWG, away from the perception that women and girls are only at risk of unknown soldiers and militias. It also has implications for how the household space is approached and responded to during peace processes. VAWG interventions within conflict-affected settings should not neglect the household sphere and interpersonal relationships, for it remains the space most dangerous to women.

The programme led to *significantly more equitable gender attitudes at endline*. This included all survey items related to gender equality, attitudes towards power in relationships, and gendered norms relating to household decision-making. There were also significantly higher stigma scores at endline, indicating a reduction in attitudes that stigmatise rape survivors. There was a *significant improvement in attitudes towards IPV*, with a significant reduction in the belief that women must tolerate violence, a significant increase in support for discussing violence, a strongly significant reduction in violence-supportive attitudes, and a significant reduction in support for sexual IPV.

Both these improvements signal the ability and influence of faith leaders and faith communities, for this was the intervention’s entry point into the communities. With the research showing the high levels of religious affiliation within the target communities, our findings confirm the appropriateness of such an entry point. There was almost universal adherence to a religion among both samples (around 95%), which was twice the rate of membership of any other community group among the baseline sample. In line with literature [e.g. 26], the reality of an almost total lack of other civil society or governmental infrastructure within these communities, again highlights the centrality of faith institutions, not only for members of the faith community, but for the community in general. *For positive attitude change was not limited to those actively engaged within faith communities*. At endline, we see a positive shift across the entire community in terms of gender attitudes, rape myths and rape stigma scores, regardless of level of faith engagement. Therefore a primary entry point of faith leaders and faith communities is reaching beyond those within their immediate circle of influence (those actively faith engaged). This speaks to the diffusion potential of messaging and training with and through faith leaders and faith communities.

This leads to two points for consideration. Firstly, the baseline data cautions against the automatic assumption that faith communities are automatically spaces promoting gender inequitable attitudes and VAWG. On the contrary, it highlights that, in the 15 intervention communities, people of faith were significantly more supportive of gender equality and non-violence, compared to the rest of the community. Secondly, it affirms the influence of faith leaders and faith communities within the broader community. Intervention messaging did not reach only those highly involved in religious activities, but also those nominally engaged, and even those not aligned with a faith group.

Our findings suggest that working with *faith leaders and faith communities as an entry point into communities is a strategic approach* and can impact the entire community. We found that very large proportion of respondents reported having participated in counselling or talks/discussions, and that such attendance had significant impact on gender equitable attitudes, more gender equitable attitudes related to masculinities, and less agreement with rape stigma. Access to counselling and teaching, not only relating to VAWG, had increased substantially over the duration of the programme, to the point that nearly two thirds of respondents in the endline sample had been reached. The trained programme actors had been especially active in providing counselling, notably faith leaders and gender champions, while an even higher proportion of respondents (83%) had attended some kind of public talk or public meeting on the topic of VAWG. The reach and impact of the activities implemented by the programme’s primary beneficiaries can arguably be seen as the reason for the increase in gender equitable attitudes in the community overall (and not only amongst those who are actively engaged in a faith community).

It should be noted that, when looking specifically at the gender of respondents, we found that, in certain cases, women were more likely to continue supporting gender inequitable attitudes at endline. For example: with the statement ‘it is manly for a man to beat his wife’, there was a significant reduction in agreement by men, but no corresponding significant reduction amongst women; and there was a significant decrease in the proportion of women agreeing that people should intervene if a man mistreats his wife. Within the qualitative panel study conducted over the course of the intervention, similar forms of complicity was seen with some of the female participants. While it is outside of the scope of this article to delve deeper into what drives this phenomenon, and without overstating the problem (for the research also showed significant positive change in women’s attitudes and beliefs), it should be noted as something that attention should be paid to within VAWG interventions in general, and that needs further research.

This research has some limitations. There is no non-exposed comparison arm and so we have no way of being confident that the findings can be attributed to the intervention rather than other temporal changes in the community, such as generally greater peacefulness or other ill-defined temporal changes. However, conflict broke out in the study area within 2 weeks of the end of endline data collection, which underlined the fact that peace-building in DRC remains work in progress. Our confidence in attribution is heightened by the findings that attitudes of those exposed to the intervention were more gender equitable than those who were not. The fact that levels of intervention exposure were very high enhances the plausibility that the intervention was effective. We know the samples at endline and baseline were not identical, but we have tested whether these differences could alone have caused the changes found at endline and we have showed that they were not the explanation. We cannot rule out the role of chance in the differences between the two measures, but the fact that these were two large samples reduces the likelihood of chance being solely the explanation. Furthermore, the fact that the samples were independent enhances our confidence that there was no effect on endline caused by respondents having participated in a questionnaire at baseline.

## Conclusion

This intervention was premised on the assumption that faith leaders and faith communities are a key entry point into an entire community, able to engage and advocate for ending VAWG, and thereby influence an entire community. Research has affirmed this assumption. A structured intervention strategy, that systematically engages with, trains and mentors faith leaders and faith community members, has been shown to impact all aspects of IPV and lead to significantly more equitable gender attitudes. However, while we are confident of the link between the social norms change and faith engagement and project exposure, the link between IPV reduction and faith engagement and project exposure needs more research.

Working with faith communities reaches not only members of the faith community, but the community in general – highlighting the strategic nature of working with and through faith communities. Especially in the 15 communities in Ituri province, where continuous conflict means that state infrastructure is largely lacking, faith communities are the most present and influential network. There are many similar communities in the world, where VAWG is common and the fragile state is unable to respond. This calls for greater engagement by VAWG actors with faith leaders and faith communities.

## Data Availability

The datasets supporting the conclusions of this article is(are) available in the South African Medical Research Council repository https://medat.samrc.ac.za/index.php/catalog/30/get_microdata

## Abbreviations

CAG: Community Action Group
DRC: Democratic Republic of Congo
IMAGES: International Men and Gender Equality Survey
IPV: Intimate Partner Violence
NPSV: Non-Partner Sexual Violence
SGBV: Sexual- and Gender-Based Violence
SVAW: Sexual Violence Against Women
ToC: Theory of Change
UK: United Kingdom
VAWG: Violence Against Women and Girls
WHO: World Health Organisation

## Footnotes

1 For more details on Tearfund’s faith-based approach to addressing harmful gender norms, and to access the training materials used, please see: https://learn.tearfund.org/en/themes/sexual_and_gender-based_violence/resources_and_publications/transforming_masculinities/

2 For instance, one of these items stated ‘a man should provide for his family’. Although the item was designed to test whether respondents supported traditional gender roles, with agreement with the statement expected to indicate support for traditional/conservative gender norms, disagreement with the statements would not necessarily correlate with more equitable gender attitudes.

3 The endline survey made a distinction between respondents who were married, those who were cohabiting and those in a girlfriend/boyfriend relationship, which is why baseline and endline figures are not aligned for all response categories.
